# Supplementary figures and images for: Ontogenetic progression of individual head size in the larvae of the beetle Trypoxylus dichotomus (Coleoptera: Scarabaeidae): catch-up growth within stages and per-stage growth rate changes across stages
Source: PeerJ. 2023 May 31;11:e15451. doi: 10.7717/peerj.15451 (PMC10239228; doi:10.7717/peerj.15451)

Supplementary fig. S1

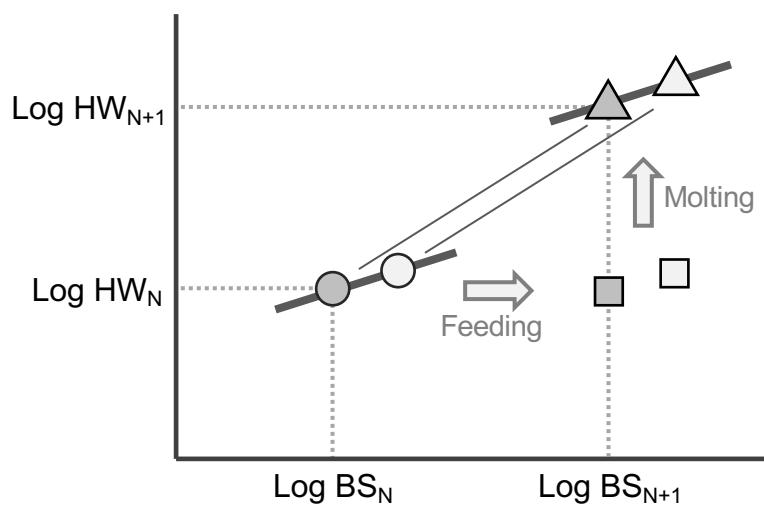

Supplement: Supplemental Information 1 — The head and body sizes of two hypothetical individuals are shown. Dark and light grey symbols represent small and large individuals, respectively. Circles and rectangles indicate the beginning and end, respectively, of instar N. Triangles indicate the beginning of instar N+1. The body size (BS) increases during the food-taking period of the instar, while the head width (HW) increases at the ecdysis from that instar to the subsequent instar. Thick and thin lines indicate the static and ontogenetic allometries, respectively. Log-transformed measurements for the HW and BS (the cubic root of body mass) at the beginning of the instar were used to calculate the allometric relationships. Raw individual measurements of HW and BS were used to calculate the individual per-stage growth rates of head width (iPSGRH) and body mass (iPSGRB). [file peerj-11-15451-s001.pdf]
